# Supplementary material for: Development strategy of early childhood music education industry: An IFS-AHP-SWOT analysis based on dynamic social network
Source: PLoS One. 2024 Feb 29;19(2):e0295419. doi: 10.1371/journal.pone.0295419 (PMC10903847; doi:10.1371/journal.pone.0295419)
Supplement: S1 File — (ZIP) [file pone.0295419.s001.zip › Supporting Information/Appendix.docx]

**Appendix**

**Appendix A**: Survey on the Development of Early Childhood Music Education Industry

Table 10 Survey on the Development of Early Childhood Music Education Industry

| Survey on the Development of Early Childhood Music Education Industry | | | | | | | | | | | | | | | | | |
| --- | --- | --- | --- | --- | --- | --- | --- | --- | --- | --- | --- | --- | --- | --- | --- | --- | --- |
| We sincerely appreciate you taking the time to participate in this survey on the early childhood music education industry. Your opinions and suggestions will have a profound impact on our understanding of this industry and its future direction.  The purpose of this survey is to understand the current status of the early childhood music education industry, the challenges it faces, and opportunities for future development. Through your genuine feedback, we hope to collect firsthand data to better understand the industry and develop targeted improvement strategies. We value the feedback from every participant, whether you are a practitioner in early childhood music education, an educational researcher, a parent, or a concerned party. We sincerely invite you to share your views and suggestions.  Rest assured, all survey information will be strictly confidential and used solely for research purposes. This survey will take about 10 minutes to complete. Thank you again for your participation and support. Your valuable feedback will make a significant contribution to the development of the early childhood music education industry.  Thank you for your time and assistance! | | | | | | | | | | | | | | | | | |
| Are you familiar with the early childhood music education industry? | | | | | | | | | | | | | | | | | |
| A. Yes | | | | | | | | | | B. No | | | | | | | |
| Do you have a child receiving music education? | | | | | | | | | | | | | | | | | |
| A. Yes | | | | | | | | | | B. No | | | | | | | |
| Are you working in the early childhood music education industry? | | | | | | | | | | | | | | | | | |
| A. Yes | | | | | | | | | | B. No | | | | | | | |
| If you chose that you work in the early childhood music education industry, please select your role(s) (multiple selections allowed): | | | | | | | | | | | | | | | | | |
| A. Music Teacher | | | | | | | | | | B. Teaching Assistant | | | | | | | |
| C. Education Management/Administration | | | | | | | | | | D. Art Director/Consultant | | | | | | | |
| E. Other (please specify): _______ | | | | | | | | | | | | | | | | | |
| Do you follow the development trends of the early childhood music education industry? | | | | | | | | | | | | | | | | | |
| A. Yes | | | | | | | | | | B. No | | | | | | | |
| Have you noticed an increase in the number of early childhood music education institutions in recent years? | | | | | | | | | | | | | | | | | |
| A. Not at all | B. Slightly | | C. Somewhat | | | | | | | D. Very much | | | | | | E. Absolutely | |
| How familiar are you with the recognition level of the early childhood music education industry? | | | | | | | | | | | | | | | | | |
| A. Very knowledgeable | B. Somewhat knowledgeable | | | | | | | | | C. Not knowledgeable | | | | | | | |
| Do you believe that the market for early childhood music education has broad prospects for development? | | | | | | | | | | | | | | | | | |
| A. Strongly disagree | B. Disagree | | C. Neutral | | | | | | | D. Agree | | | | | | E. Strongly agree | |
| What is your view on the competition in the early childhood music education industry? | | | | | | | | | | | | | | | | | |
| A. Intense | B. Moderate | | | | | | | | | C. Not intense | | | | | | | |
| How significant do you think the impact of music education is on a child's overall development? | | | | | | | | | | | | | | | | | |
| A. Very Small | B. Somewhat Small | | C. Moderate | | | | | | | D. Somewhat Large | | | | | | E. Very Large | |
| What are the reasons for your child's participation in early childhood music education? (multiple selections allowed) | | | | | | | | | | | | | | | | | |
| A. Improving emotional expression and communication skills | | | | | | | | B. Improving cognitive skills | | | | | | | | | |
| C. Cultivating interest and appreciation in music | | | | | | | | D. Developing creativity and imagination | | | | | | | | | |
| E. Enhancing social skills and teamwork | | | | | | | | F. Expanding subject knowledge and skills | | | | | | | | | |
| G. Other (please specify): _______ | | | | | | | | | | | | | | | | | |
| Do you think the teaching content of early childhood music education is keeping up with the times? | | | | | | | | | | | | | | | | | |
| A. Yes | | | | | | | | | B. No | | | | | | | | |
| What percentage of your total education budget is invested in early childhood music education? | | | | | | | | | | | | | | | | | |
| A.0%-20% | B.20%-40% | | C. 40%-60% | | | | | | | | D. 60%-80% | | | | E. 80%-100% | | |
| Has your child ever participated in any music competitions or public performances organized by music education institutions? | | | | | | | | | | | | | | | | | |
| A. Yes | | | | | | | B. NO | | | | | | | | | | |
| Are you satisfied with the teaching quality of early childhood music education institutions? | | | | | | | | | | | | | | | | | |
| A. Extremely satisfied | B. Satisfied | C. Neutral | | | | | | | | D. Dissatisfied | | | | | | | E. Extremely dissatisfied |
| Do you think the early childhood music education industry should integrate more technology into teaching? | | | | | | | | | | | | | | | | | |
| A. Yes | | | | | | | B. No | | | | | | | | | | |
| Are you willing to continue to support your child's participation in early childhood music education? | | | | | | | | | | | | | | | | | |
| A. Yes | | | | | | | B. No | | | | | | | | | | |
| How satisfied are you with the services provided by the early childhood music education institutions? | | | | | | | | | | | | | | | | | |
| A. Not at all | B. Slightly | C. Somewhat | | | | | | | | | | | | D. Very much | | | E. Absolutely |
| Would you recommend early childhood music education to other parents? | | | | | | | | | | | | | | | | | |
| A. Yes | | | | | | B. No | | | | | | | | | | | |
| In which areas do you believe the early childhood music education industry needs to improve? (multiple selections allowed) | | | | | | | | | | | | | | | | | |
| A. Quality and content of teaching | | | | | B. Teacher quality and training | | | | | | | | | | | | |
| C. Teaching facilities and resources | | | | | D. Education management and services | | | | | | | | | | | | |
| E. Pricing and cost issues | | | | | F. Other (please specify): _______ | | | | | | | | | | | | |
| In what areas should early childhood music education institutions provide more support and help? (multiple selections allowed) | | | | | | | | | | | | | | | | | |
| A. Provide tuition discounts | | | | | B. Provide course recommendations and planning | | | | | | | | | | | | |
| C. Provide educational training and lectures | | | | | D. Provide an online learning platform | | | | | | | | | | | | |
| E. Provide progress tracking | | | | | F. Other (please specify): _______ | | | | | | | | | | | | |
| Do you think the teaching materials and tools in the early childhood music education industry are diverse and abundant? | | | | | | | | | | | | | | | | | |
| A. Yes | | | | | B. No | | | | | | | | | | | | |
| Are you satisfied with the complaint handling mechanism in the early childhood music education industry? | | | | | | | | | | | | | | | | | |
| A. Extremely satisfied | B. Satisfied | C. Neutral | | | | | | | | | | D. Dissatisfied | | | | | E. Extremely dissatisfied |
| Would you like to learn about the training and learning resources in the early childhood music education industry? | | | | | | | | | | | | | | | | | |
| A. Yes | | | | | | B. No | | | | | | | | | | | |
| Are you satisfied with the promotion and marketing methods of the early childhood music education industry? | | | | | | | | | | | | | | | | | |
| A. Extremely satisfied | B. Satisfied | | | C. Neutral | | | | | | | | | D. Dissatisfied | | | | E. Extremely dissatisfied |
| Do you believe the early childhood music education industry should conduct more parent-child education activities? | | | | | | | | | | | | | | | | | |
| A. Yes | | | | | | | | B. No | | | | | | | | | |
| Would you be willing to have your child participate in extracurricular activities for early childhood music education? | | | | | | | | | | | | | | | | | |
| A. Yes | | | | | | | | | B. No | | | | | | | | |
| In which areas do you believe the early childhood music education industry should strengthen its improvements? (multiple selections allowed) | | | | | | | | | | | | | | | | | |
| A. Enhance teacher training and professionalism | | | | | | | | | | B. Expand teaching resources and facilities | | | | | | | |
| C. Increase the application of tech in teaching | | | | | | | | | | D. Enhance the diversity and quality of courses | | | | | | | |
| E. Improve tuition fees and discount policies | | | | | | | | | | F. Strengthen communication and cooperation with parents. | | | | | | | |
| G. Other (please specify): _______ | | | | | | | | | | | | | | | | | |

**Appendix B**: Policies and Regulations

Table 11 Policies and Regulations

| "Opinions on Deepening Education and Teaching Reforms to Improve the Quality of Compulsory Education" |
| --- |
| **Policy Support:** This policy strictly implements courses such as music, art, and calligraphy, establishes special art courses combined with local culture, and conducts widespread campus art activities, guiding students to understand outstanding world art. It supports art schools in establishing support bases in primary and middle schools.  **Policy restrictions:** It establishes an evaluation system guided by quality education, setting evaluation standards for the quality of compulsory education, school education quality, and student development quality. The evaluation mainly focuses on the school's comprehensive training capabilities and the development of students' morality, academics, and health. It continuously improves the compulsory education quality monitoring system, focusing on process-oriented and developmental evaluations, setting up a monitoring platform, and regularly publishing reports. |
| "Opinions of the Central Committee of the Communist Party of China and the State Council on Deepening the Reform of Teacher Team Building in the New Era" |
| **Policy Support:** This policy aims to comprehensively strengthen the construction of teachers' ethics and style, vigorously revitalise teacher education, continuously enhance professional competencies, deepen the comprehensive reform of teacher management, straighten out the system mechanism, continuously improve status and treatment, truly making teaching an enviable profession, and strengthen the leadership of the party, to ensure that policy measures are effective.  **Policy restrictions:** This policy perfects the title evaluation standards, emphasizing the integration of morality and talent, and teaching achievements to inspire teachers' vitality. It avoids formalistic assessments interfering with teaching and doesn't simply evaluate teachers based on college entrance rates or exam results. It implements a regular registration system, establishes a teacher exit mechanism, and enhances team vitality. It also strengthens the assessment of primary and secondary school principals, improves their abilities, and perfects the survival-of-the-fittest mechanism. |
| "Overall Plan for Deepening the Reform of Education Evaluation in the New Era" |
| **Policy Support:** The policy aims to improve the evaluation of aesthetic education. It incorporates the learning of arts subjects such as music, fine arts, calligraphy, etc., as well as participation in school-organized art practice activities into academic requirements for primary and secondary school students. The goal is to promote students' artistic interests, enhance their artistic literacy, and fully improve their abilities to perceive, express, appreciate, and create beauty. The policy also explores the inclusion of arts subjects in the reform pilot of the high school entrance examination. It encourages universities to include public art courses and art practice into talent training programs, implement a credit system, and stipulate that students can only graduate after completing the required credits.  **Policy restrictions:** The policy establishes a diversified evaluation system, which is uniformly monitored by the education supervision department, with professional organizations and social organizations playing their roles. It aims to control the number and frequency of evaluation activities to reduce the burden on grassroots and schools. It innovates teaching research guidance methods and restricts assessment by examination. Using artificial intelligence and big data, it explores comprehensive vertical and horizontal evaluation throughout the process. It improves the use of evaluation results, strengthens the capacity building of teacher evaluation, supports the establishment of related majors, and cultivates specialized talents. It also strengthens the construction of the national education examination team and perfects the teacher incentive mechanism. |
| "Opinions on Deepening Educational and Teaching Reforms to Fully Improve the Quality of Compulsory Education" |
| **Policy Support:** This policy emphasizes the "five educations" (moral education, intellectual education, physical education, aesthetic education, and labor education), aiming for the comprehensive development of quality education. It earnestly implements the new requirements of the Party Central Committee and the State Council on "developing quality education", enhances the respective status of moral, physical, aesthetic, and labor education, highlights the practical effects of moral education, raises the level of intellectual education, strengthens physical exercise, enhances aesthetic edification, strengthens labor education, and promotes the all-round development of students.  **Policy restrictions:** The policy calls for the improvement of the quality evaluation and monitoring system, the establishment of a quality education-oriented evaluation system, and the formulation of evaluation standards for the quality of compulsory education, school quality, and student development. The evaluation includes the support for educational reform from localities, the comprehensive training of schools, and the moral development of students. It strengthens the monitoring of compulsory education quality, emphasizing process-oriented and developmental evaluation, setting up a monitoring platform, and regularly releasing reports. |
| "Opinions on Further Reducing the Burden of Homework and Off-Campus Training for Students in Compulsory Education Stage" |
| **Policy Support:** This policy establishes a supervision system and training material management methods, prohibiting over-standard training, non-disciplinary organizations conducting subject training, and the provision of overseas courses. It investigates and deals with over-range training, quality problems, illegal content, and infringement actions. According to the Protection of Minors Act, training institutions are prohibited from occupying holidays and vacation time, as well as offering high salaries to school teachers. Subject training personnel must possess teacher qualifications and make them public, while also protecting the information of parents and students. Fees are determined based on the market and costs, and are publicly disclosed for supervision.  **Policy restrictions:** Each region strictly reviews and registers non-profit extracurricular subject training institutions, prohibits financing and foreign capital control, and strengthens supervision of online training institutions. Institutions must publicly disclose teacher qualification information, protect personal information, and be open and transparent in charging fees. Online training is limited to 30 minutes per lesson, with a 10-minute break in between, and must end by 9pm. It is prohibited to provide learning methods that affect students' thinking ability. Unfair competition and industry monopoly are strictly forbidden. |

**Appendix C**: Comparative analysis

We validate the robustness and effectiveness of the methods used in this paper from two perspectives. One is to revalidate the cases in this paper using the methods from relevant literature, and the other is to validate the cases from the relevant literature using the methods presented in this paper. However, different evaluation values are used in different papers. For instance, Dağdeviren and Yüksel (2011) used linguistic fuzzy numbers, Papapostolou et al. (2020) used triangular fuzzy numbers, and Tavana et al. (2016) used triangular intuitionistic fuzzy numbers. Therefore, it is necessary to unify these evaluation values into a common fuzzy number evaluation using the following methods:

1. Conversion of Different Fuzzy Number Evaluation Values

Let the intuitionistic fuzzy number be represented as $a=\left( \mu_{a},\nu_{a} \right)$; the triangular intuitionistic fuzzy number be represented as $b=\left\langle\left( \underline{b},b,\overline{b} \right);\delta_{b},\varepsilon_{b} \right\rangle$, where $\delta_{b}$ represents the maximum membership degree, and $\varepsilon_{b}$ represents the minimum non-membership degree. The conversion relationship between the intuitionistic fuzzy number $a$ and the triangular intuitionistic fuzzy number $b$ is shown in formulas 24-25:

| $\mu_{a}=\left\{ \begin{aligned} {\left( x-\underline{b} \right)\delta_{b}}/\left( b-\underline{b} \right) if \underline{b}\leq x<b \\ \delta_{b} if x=b \\ {\left( \overline{b}-x \right)\delta_{b}}/\left( \overline{b}-b \right) ifb<x\leq\overline{b} \\ 0 if x<\underline{b} or x>\overline{b} \end{aligned} \right.$ | (24) |
| --- | --- |
| $\nu_{a}=\left\{ \begin{aligned} \left( b-x+\left( x-\underline{b} \right)\delta_{b} \right)/\left( b-\underline{b} \right) if \underline{b}\leq x<b \\ \varepsilon_{b} if x=b \\ \left( x-b+\left( \overline{b}-x \right)\delta_{b} \right)/\left( \overline{b}-b \right) ifb<x\leq\overline{b} \\ 1 if x<\underline{b} or x>\overline{b} \end{aligned} \right.$ | (25) |

The conversion relationships between intuitionistic fuzzy numbers, linguistic fuzzy numbers, and triangular fuzzy numbers are shown in Table 11 (Wu and Xu, 2015; Eftekhary et al., 2012):

Table 11. Conversion Table for Indicator Evaluation Linguistic Term Sets

| Linguistic fuzzy terms | Triangular fuzzy numbers | Intuitive fuzzy numbers |
| --- | --- | --- |
| Very Good/Very High | (9,10,10) | (0.85,0.1) |
| Good/High | (7,9,10) | (0.75,0.15) |
| Fairly Good/Fairly High | (5,7,9) | (0.65,0.25) |
| Moderate/Medium | (3,5,7) | (0.5,0.4) |
| Fairly Poor/Fairly Low | (1,3,5) | (0.35,0.55) |
| Poor/Low | (0,1,3) | (0.25,0.65) |
| Very Poor/Very Low | (0,0,1) | (0.15,0.8) |

2. Use the methods in related papers to re-verify the case in this Paper

|  | S | W | O | T |
| --- | --- | --- | --- | --- |
| This Paper | 1.5076 | 0.9168 | 0.946 | 0.7837 |
| Dağdeviren and Yüksel (2011) | 1.2538 | 1.1404 | 0.7231 | 0.7324 |
| Papapostolou et al., 2020 | 1.2647 | 1.3844 | 1.212 | 0.9464 |
| Tavana et al., 2016 | 1.1421 | 1.005 | 0.848 | 0.7197 |

3. Use the method in this Paper to verify the cases in related papers

| The original result of Dağdeviren and Yüksel (2011) | Work system 1 has a score of 0.557 and Work system 2 has a score of 0.372. Work system 1 is better than Work system 2. |
| --- | --- |
| The result of Dağdeviren and Yüksel (2011) verified by the method of this Paper | Work system 1 has a score of 0.742 and Work system 2 has a score of 0.531. Work system 1 is better than Work system 2. |
| The original result of Papapostolou et al., 2020 | SO strategy $\succ$ ST strategy $\succ$ WO strategy $\succ$ WT strategy |
| The result of Papapostolou et al., 2020 verified by the method of this Paper | SO strategy $\succ$ WO strategy $\succ$ ST strategy $\succ$ WT strategy |
| The original result of Tavana et al., 2016 | The strengths more important than weaknesses, opportunities, and threats. |
| The result of Tavana et al., 2016 verified by the method of this Paper | Strengths and opportunities are almost equally important, but both are far more important than weaknesses and threats. |

From the above results, it can be observed that whether revalidating the cases in this paper using methods from relevant literature or validating the cases from relevant literature using the methods presented in this paper, the final results, although with slight variations, do not exhibit significant changes. This indicates the robustness and effectiveness of the methods employed in this paper across different cases and evaluation values.

Wu, Z., & Xu, J. (2015). Possibility distribution-based approach for MAGDM with hesitant fuzzy linguistic information. *IEEE transactions on cybernetics*, *46*(3), 694-705.

Eftekhary, M., Safari, S., Shojaee, M., Assarian, M., & Karimi, I. (2012). Identifying customers needs on electronic services of bank using fuzzy QFD approach. *Aust. J. Basic Appl. Sci*, *6*, 287-296.

Dağdeviren, M., & Yüksel, İ. (2008). Developing a fuzzy analytic hierarchy process (AHP) model for behavior-based safety management. *Information sciences*, *178*(6), 1717-1733.

Papapostolou, A., Karakosta, C., Apostolidis, G., & Doukas, H. (2020). An AHP-SWOT-Fuzzy TOPSIS approach for achieving a cross-border RES cooperation. *Sustainability*, *12*(7), 2886.

Tavana, M., Zareinejad, M., Di Caprio, D., & Kaviani, M. A. (2016). An integrated intuitionistic fuzzy AHP and SWOT method for outsourcing reverse logistics. *Applied soft computing*, *40*, 544-557.

**Appendix D**:

Example 1: Suppose there are two intuitionistic fuzzy numbers$a=\left( 0.2,0.3 \right)$ and $b=\left( 0.4,0.1 \right)$,

$$dis\left( a,b \right)=\frac{\left( \left| \mu_{a}-\mu_{b} \right|+\left| \nu_{a}-\nu_{b} \right| \right)}{2}=\frac{\left( \left| 0.2-0.4 \right|+\left| 0.3-0.1 \right| \right)}{2}=0.2$$

Example 2: Suppose there are two intuitionistic fuzzy numbers$a=\left( 0.2,0.3 \right)$ and $b=\left( 0.4,0.1 \right)$,

$$\rho\left( a \right)=0.5\left( 1+\left( 1-0.2-0.3 \right) \right)\left( 1-0.2 \right)=0.6$$

$$\rho\left( b \right)=0.5\left( 1+\left( 1-0.4-0.1 \right) \right)\left( 1-0.4 \right)=0.45$$

so, $b\succ a$

Example 2: Suppose there are two intuitionistic fuzzy numbers$a=\left( 0.2,0.3 \right)$ and $b=\left( 0.4,0.1 \right)$, $\lambda=0.5$

$$a\bigoplus b=\left( 0.2+0.4-0.2\times0.4,0.3\times0.1 \right)=\left( 0.52,0.03 \right)$$

$$a\bigotimes b=\left( 0.2\times0.4,0.3+0.1-0.3\times0.1 \right)=\left( 0.08,0.37 \right)$$

$$\lambda a=\left( 1-\left( 1-0.2 \right)^{0.5},\left( 0.3 \right)^{0.5} \right)=\left( 0.11,0.55 \right)$$

$$a^{\lambda}=\left( \left( 0.2 \right)^{\lambda},1-\left( 1-0.3 \right)^{\lambda} \right)=\left( 0.45,0.16 \right)$$

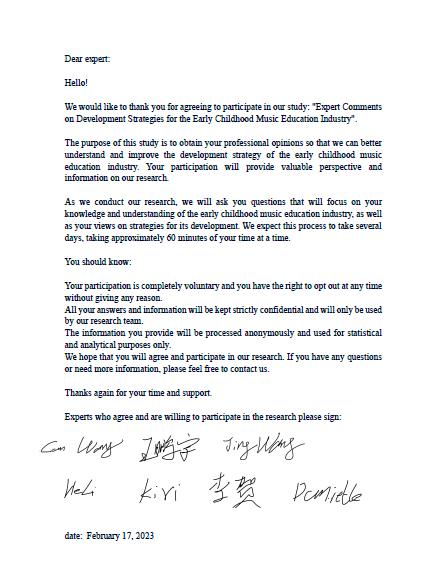


Figure 10 Informed consent form
