# Supplementary figures and images for: Development strategy of early childhood music education industry: An IFS-AHP-SWOT analysis based on dynamic social network
Source: PLoS One. 2024 Feb 29;19(2):e0295419. doi: 10.1371/journal.pone.0295419 (PMC10903847; doi:10.1371/journal.pone.0295419)

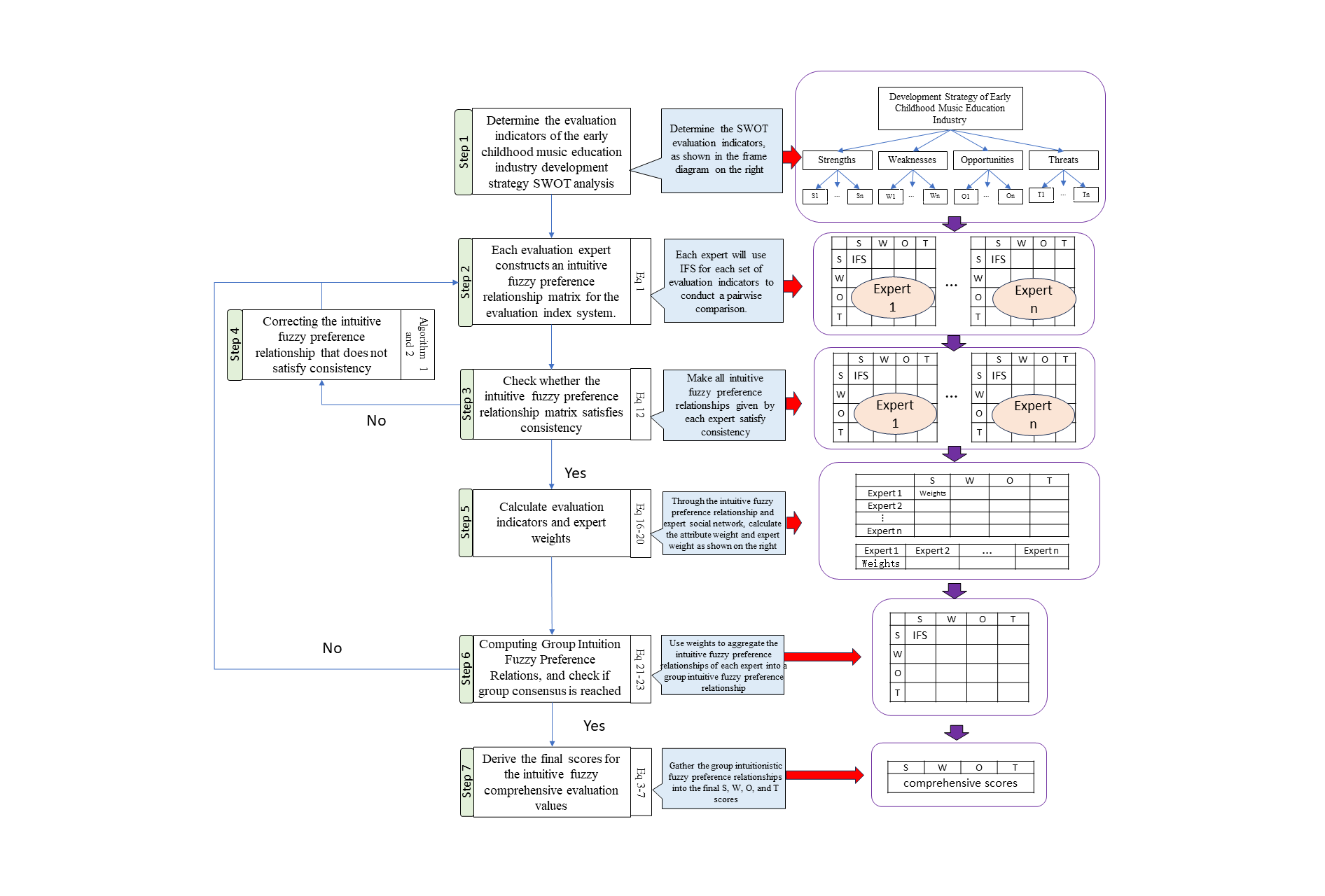

Supplement: S1 File — (ZIP) [file pone.0295419.s001.zip › Supporting Information/figures/Figure 1. Decision-making steps flow chart.tif]

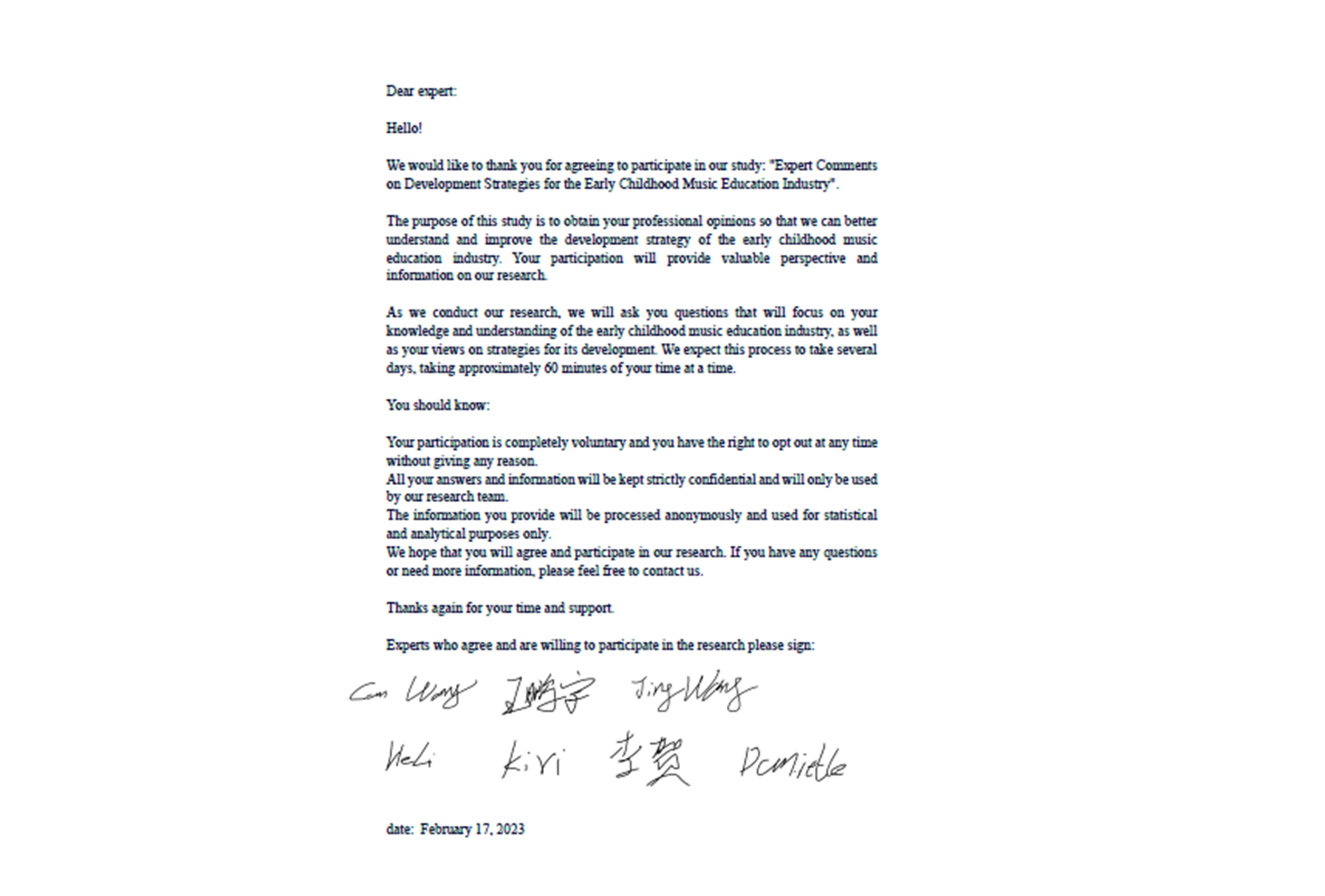

Supplement: S1 File — (ZIP) [file pone.0295419.s001.zip › Supporting Information/figures/Figure 10 Informed consent form.tif]

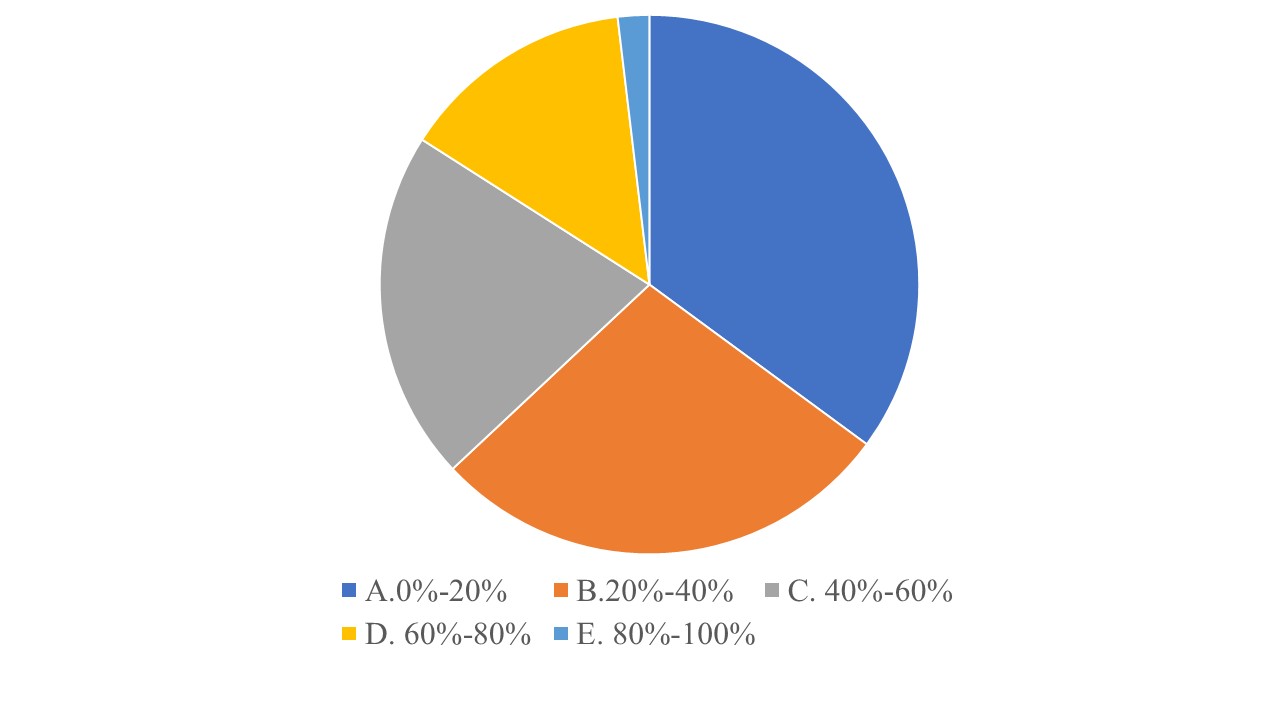

Supplement: S1 File — (ZIP) [file pone.0295419.s001.zip › Supporting Information/figures/Figure 2. Budget Allocation for Early Childhood Music Education.tif]

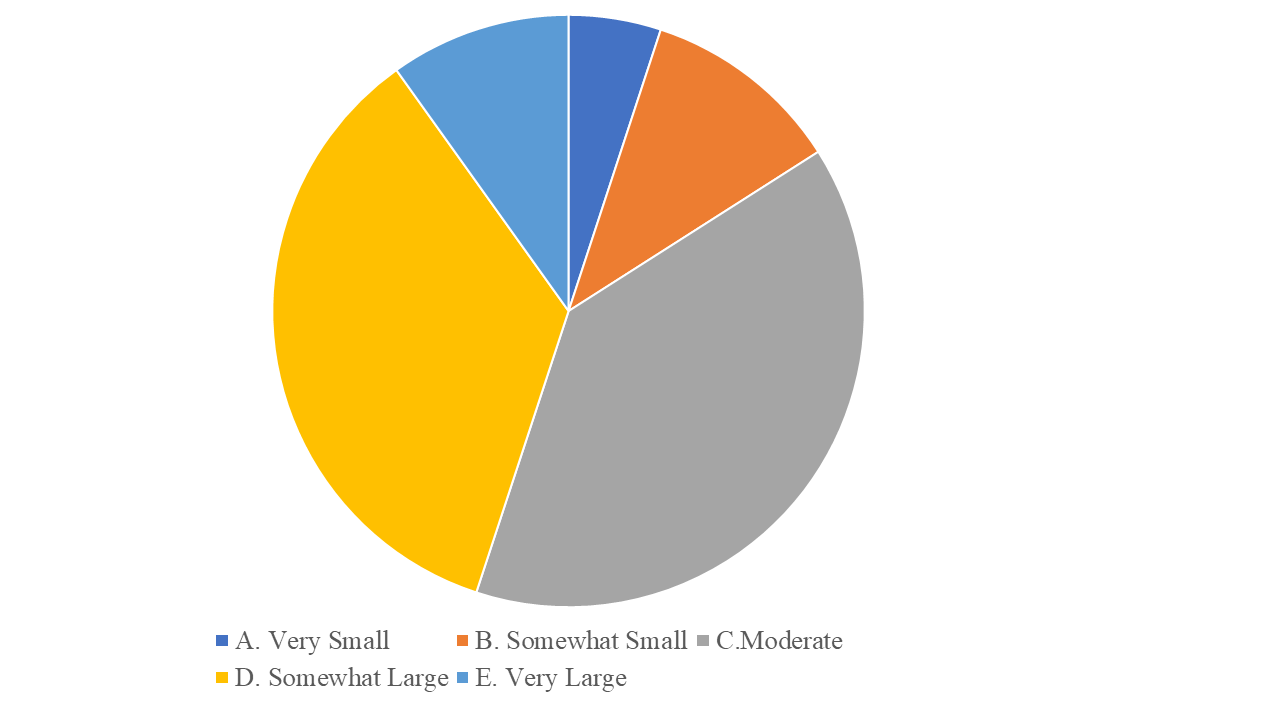

Supplement: S1 File — (ZIP) [file pone.0295419.s001.zip › Supporting Information/figures/Figure 3.Perceived Impact of Music Education on Childí»s Development.tif]

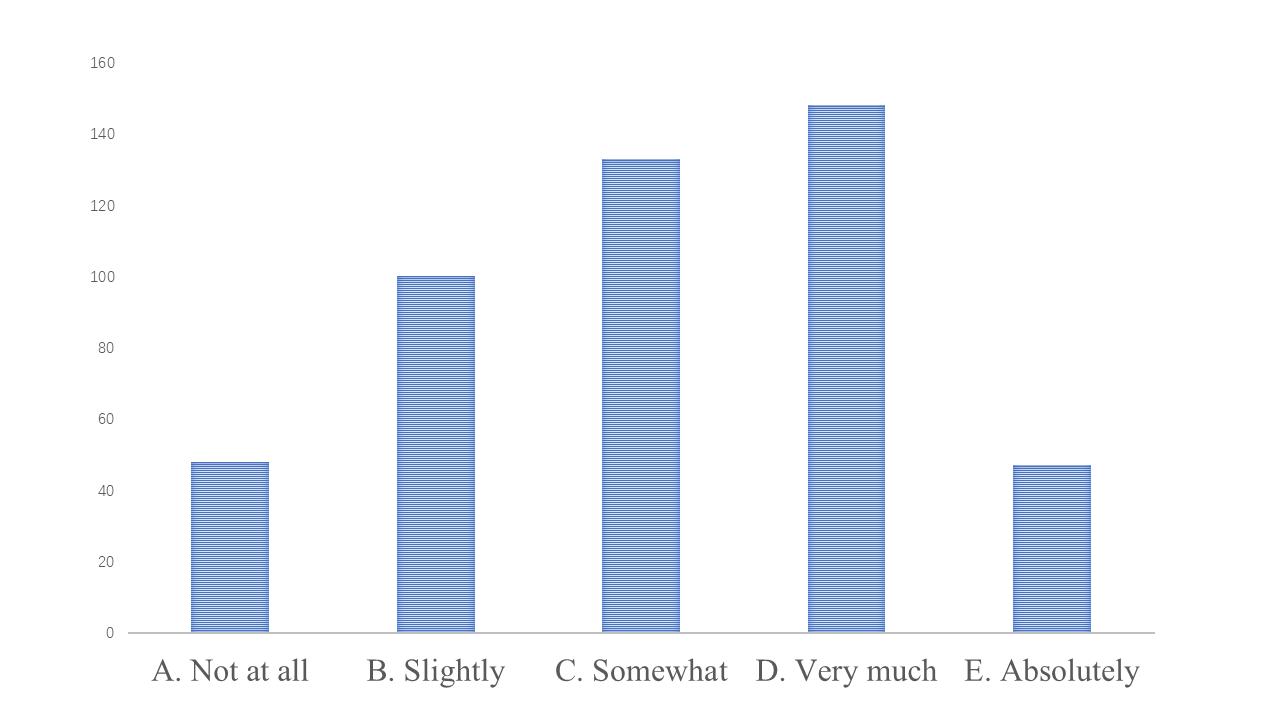

Supplement: S1 File — (ZIP) [file pone.0295419.s001.zip › Supporting Information/figures/Figure 4. Perception of the Increase in Early Childhood Music Education Institutions.tif]

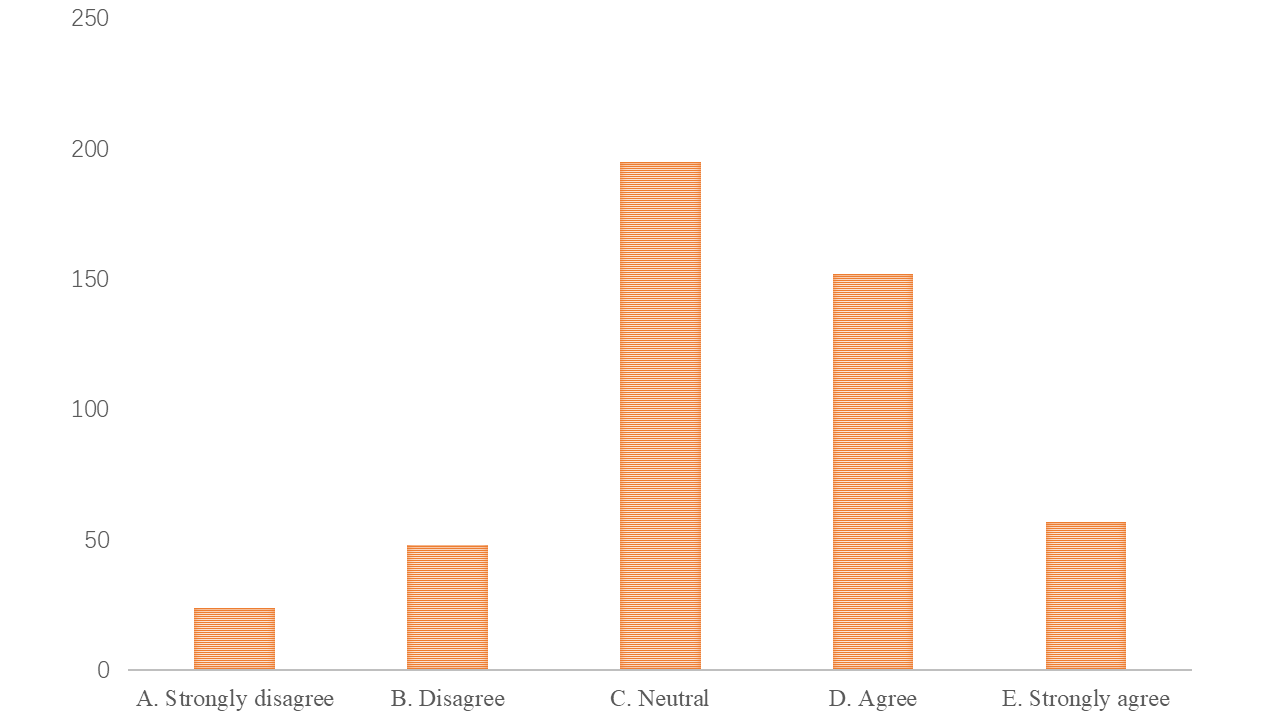

Supplement: S1 File — (ZIP) [file pone.0295419.s001.zip › Supporting Information/figures/Figure 5. Belief in the Growth Prospects of Early Childhood Music Education Market.tif]

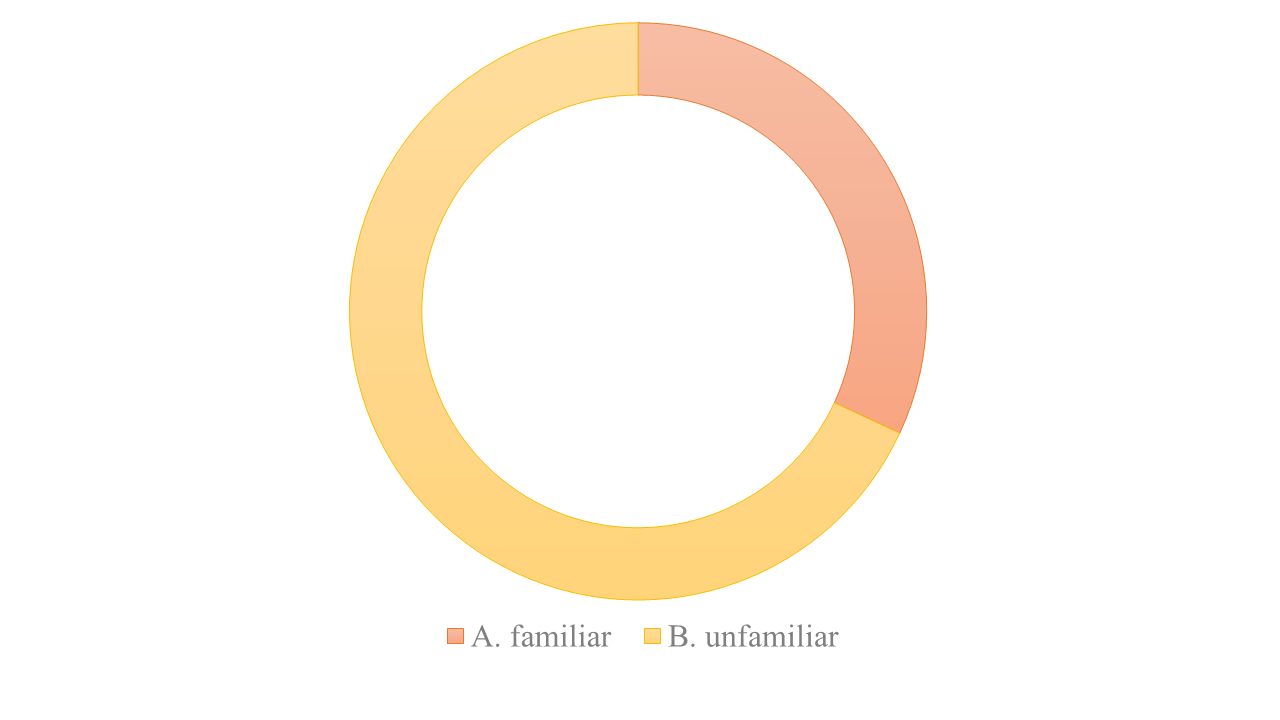

Supplement: S1 File — (ZIP) [file pone.0295419.s001.zip › Supporting Information/figures/Figure 6. Respondents' Familiarity with Well-known Early Childhood Music Education Institutions.tif]

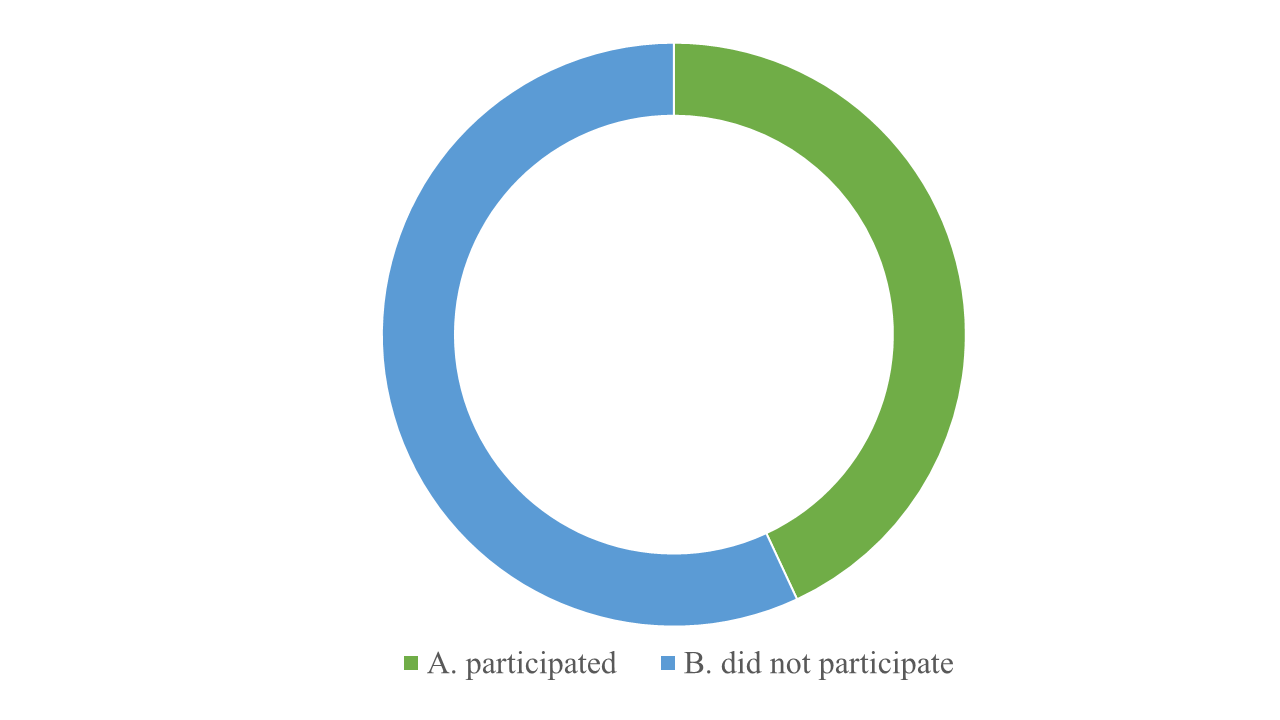

Supplement: S1 File — (ZIP) [file pone.0295419.s001.zip › Supporting Information/figures/Figure 7. Participation of Respondents' Children in Music Education Activities.tif]

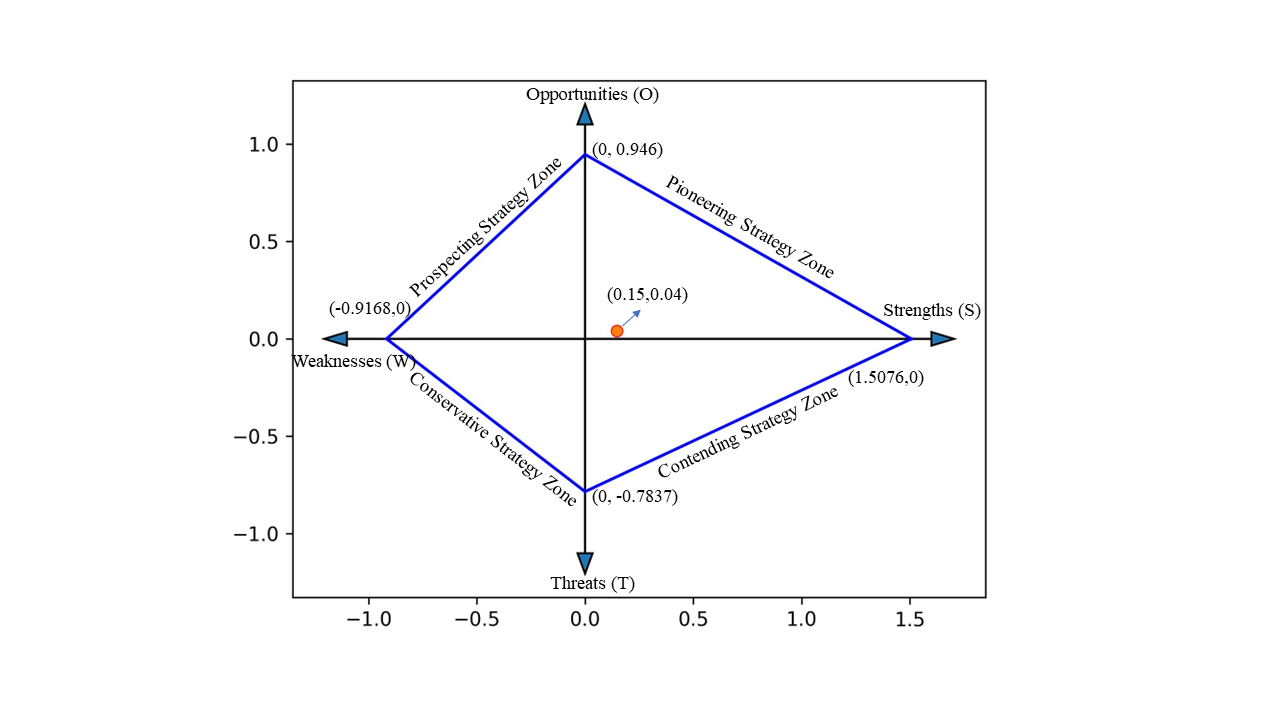

Supplement: S1 File — (ZIP) [file pone.0295419.s001.zip › Supporting Information/figures/Figure 8. Development Strategy Quadrilateral for Early Childhood Music Education Industry.tif]

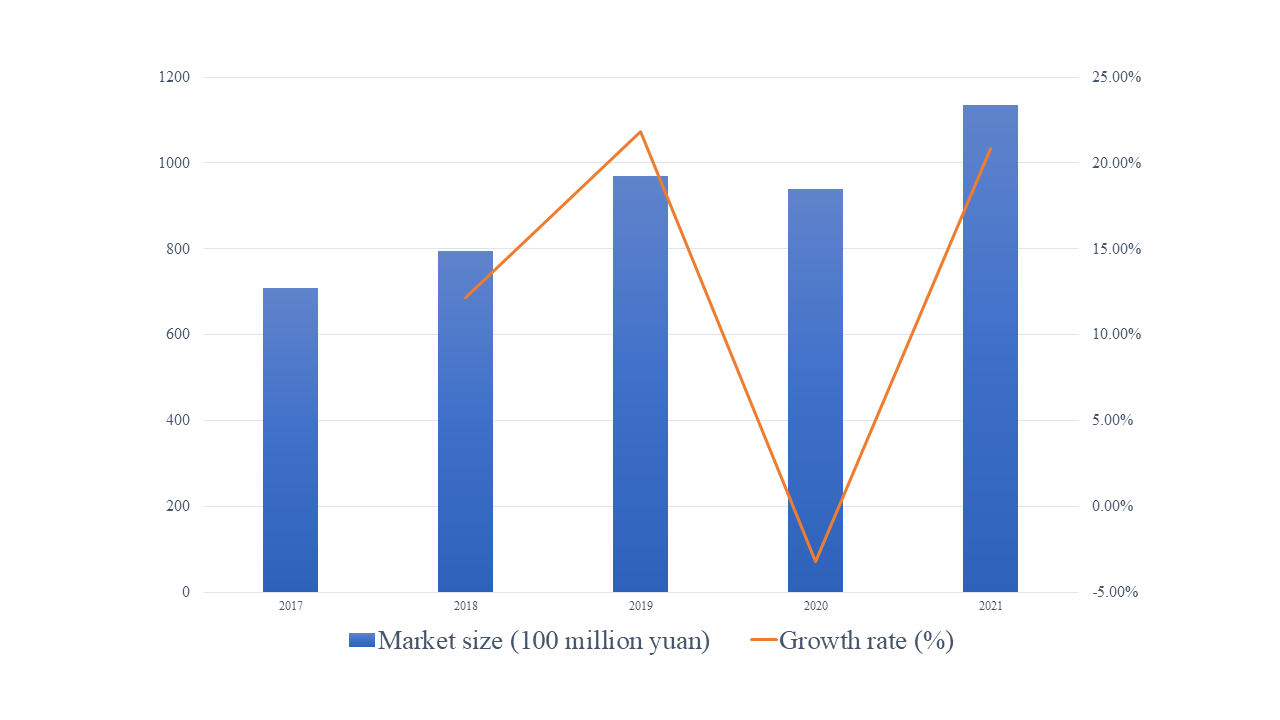

Supplement: S1 File — (ZIP) [file pone.0295419.s001.zip › Supporting Information/figures/Figure 9. Changes in market size of Chinaí»s music education industry from 2017 to 2021.tif]
